# Visual Selection: Usually Fast and Automatic; Seldom Slow and Volitional; A Reply to Commentaries

**DOI:** 10.5334/joc.32

**Published:** 2018-05-14

**Authors:** Jan Theeuwes

**Affiliations:** 1 Vrije Universiteit Amsterdam, NL

**Keywords:** Attention, Visual search, Cognitive Control

## Abstract

In this reply I react to the commentaries of my colleagues to my review article “Visual Selection: Usually Fast and Automatic; Seldom Slow and Volitional” (Theeuwes, 2018). I have organized the reply into separate sections and discuss issues that were commonly raised.

With great pleasure I read the commentaries of my colleagues to my review article “*Visual Selection: Usually Fast and Automatic; Seldom Slow and Volitional*” (Theeuwes, 2018). I would like to thank them for their excellent comments and suggestions. This kind of discussion helps the field to become more precise and accurate in using definitions and descriptions. I have organized the reply to the commentators into separate sections and addressed the issues that were commonly raised. On the basis of the commentaries, I also raise some outstanding issues for future research.

## 1. Defining top-down control

The most pressing concerns were raised about the way I have defined “top-down”. Some authors like to use a “broad” category for top-down and suggest to label anything that is not perceptual (i.e., bottom-up) as “cognitive and top-down” (Egeth, 2018). Similarly, some claim that the term top-down should be used for anything that is influenced by “*context, learning, or expectation*” because this is how cognitive psychologists have traditionally described it (Gaspelin & Luck, 2018). Also, others considered my definition of top-down control to be too narrow as it focused too much on volition (Sisk, Remington & Jiang, 2018), specifically what Wolfe referred to as “*moment-to-moment acts of volition*” (Wolfe, 2018). Others called my definition too limited because they claimed that: (1) top-down control can occur without deliberate intent (Sisk et al., 2018); (2) top-down control can be involuntary (Gaspelin & Luck, 2018) and (3) top-down control does not need awareness (Gaspelin & Luck, 2018).

The arguments provided in the commentaries seem compelling and what would be wrong with using the broad category of top-down attention as the commentators prefer to adopt? Indeed, why not call any attentional process related to “*context, learning, or expectation*” top-down even when we are not aware of them, even when they occur automatically without intent and even when you cannot counteract these effects if you try? The answer is simple. If we put everything under the umbrella term top-down, the term becomes redundant. For a scientific discussion to flourish, we need precise definitions that are limited in scope. If processes are well described and defined, we can test their boundary conditions, advancing the field instead of arguing about processes that are vague and undefined and are consistent with any experimental finding.

Gaspelin and Luck argue that attentional guidance that is involuntary and unconscious could very well be top-down even if this guidance is inconsistent with the goals and task set of the observer. If we adopt such a definition, how can we distinguish these broad top-down processes and the top-down processes as I defined them in my paper (i.e., intentional, effortful and goal-directed)? It all becomes a blur in which anything that is not driven by external saliency is top-down. It brings us nowhere.

Another example to illustrate this point: Egeth referred to studies that have shown that stimuli that have been rewarded during a training phase continue to capture attention in a test phase even when they are no longer relevant for the task (Anderson, Laurent & Yantis, 2011). In his commentary, Egeth has no problem calling these effects top-down, suggesting that anything that grabs attention – even when these stimuli are not relevant for the task at hand – should be considered a top-down effect. I think this is wrong because in these reward studies as well as in the classic attentional capture studies (Theeuwes, 1991, 1992) the field (including for example Yantis and Egeth, 1999) has always provided compelling arguments that what one is looking for (i.e., top-down search goal) should be orthogonal to stimuli that have the ability to capture attention. Only then one is able to determine the contributions of these different processes.

This brings me to maybe the most crucial aspect. It would be a dead-end if selection that is inconsistent with the current goals of the observer is labeled as top-down because it is associated with “*context, learning, or expectations*”. The fact that historically selection history has been labeled as top-down (Gaspelin & Luck) is not a convincing argument. It is absolutely crucial to fix this historical inaccuracy. Indeed, it is time to start investigating the interaction between attentional guidance that is intentional, and goal-directed, guidance related to bottom-up physical salience, and guidance related to lingering selection history biases even if these biases are the result of “*context, learning, and expectations*”.

Clear definitions are needed and I was happy to see that other commentators agreed with my research plan. Wolfe (2018) writes “*It is not a bad idea to distinguish between effects based on the top-down intentions of the searcher and those based on the selection history and we are happy to agree that priming sensibly falls into the selection history category*” (p. 1). Instead of selection history, Chelazzi & Santandrea (2018) suggest the term “*experience-dependent attentional control*” and indicate that “*experience might be so effective in inducing plasticity in the brain*” (p. 1) that the effects mimic “*the neural signatures of true bottom-up effects*” (p. 1). Kryklywy and Todd (2018) take our notion a bit further and suggest that “*long-term life history plays a crucial role in tuning attention in numerous complex ways. All of these may influence the representation of features or objects within a priority map*” (p. 2).

## 2. Contingent capture/search goals

I need to briefly comment on contingent capture and search goals. As noted by Sisk et al. “*contingent capture presumes that behavioral goals establish a set that specifies which features lead to efficient attention allocation*” (p. 1). I have argued in the target paper that this can only be done as long as the goals do not change over a block of trials (Belopolsky, Schreij & Theeuwes, 2010). Sisk et al. go even a bit further and argue that according to contingent capture “*attentional control settings can occur without deliberate intent*” (p. 1) implying that “*dormant goals spontaneously intrude into ongoing activities, suggesting that goal states are not always under volitional control*”. (p. 2). This is an interesting claim, yet a claim with implications that are challenging. Indeed, imagine an experiment in which a completely irrelevant red object captures attention. One can easily claim that the goal of selecting red things “*spontaneously intruded into the ongoing activity*”. So regardless, anytime an effect is (or is not) found, it can be attributed to unobservable, unintentional changes in the goal state of the observer.

The same holds for the well-known search modes (first defined by Bacon & Egeth, 1994), which are unobservable and widely used to explain why sometimes effects are present and sometimes not. The reasoning goes like this: if in the additional singleton paradigm capture is found, observers must have used the singleton detection mode; if no capture is found, observers must have intentionally switched to the feature search. In their commentary, Gaspelin and Luck argued that these search modes are “*very much like voluntary strategies*” (Gaspelin & Luck, 2018; p. 3). This is very unlikely. Search modes are not voluntary strategies and even though an experiment like this still needs be done, observers apparently cannot switch at will from one mode to another. Remember that Leber and Egeth (2006) were only able to induce feature search after training observers for 480 trials with specific displays that induced such a search mode. This does not sound much like a *voluntary strategy* but instead seems to be a prime example of what we have called lingering biases due to history-based selection. Search modes depend on the display properties: if one has to search for a specific shape (a diamond between squares, triangle and circles) observers have to use what has been referred to as feature search. This type of search is much slower, with no or much reduced capture effects. As argued before these search modes represent nothing else than a gradual difference between parallel and (clump-wise) serial search (Theeuwes, 2004). It has nothing to do with voluntary top-down strategies, but instead it is nothing else than less efficient search induced by the display properties (Belopolsky, Zwaan, Theeuwes & Kramer, 2007).

## 3. Other issues

*Multiple maps:* In the figure of my target article, I had all signals that could bias attention acting on one priority map by means of simple linear computations (plus and minus signs). As suggested by both Chelazzi and Santandrea (2018) and Kryklywy and Todd (2018) it is feasible that there are multiple maps. I concur with the notion of multiple maps, possibly even with different context dependent weightings as suggested by Todd and Manaligod (2017). Yet, regardless how many maps exist, and what computations take place, ultimately in visual selection there has to be some form of a “master priority map” because selection depends on competition in a winner-take-all fashion. For example, this winner take-all-notion is clear when considering overt selection involving eye movements, as our eyes can only go to one location at a time. The idea brought up by Chelazzi and Santandrea that there may be different maps for different motor output systems (hands, eyes) is interesting. I agree with the commentators that studying the interaction between different priority maps, and different biases are crucial, and for that reason alone one requires the sharp definitions that I have outlined above.

*Time course:* In my target article, I stressed the fact that history-based selection is relatively fast, comparably fast to bottom-up selection by salient singletons. Consistent with this notion, Chelazzi and Santandrea point out that experience induces plasticity in the brain, changing representations in early sensory areas. At the same time, I pointed out that top-down selection (at least the way I defined top-down) is relatively slow. Wolfe points out that top-down selection does not need to be slow. Similarly, Becker (2018) points out that the evidence from eye movement studies demonstrating that saccades with the shortest latencies typically go to the salient distractor (she refers in her commentary to a study by Mulckhuyse, van Zoest & Theeuwes, 2009), may not be that convincing. She even claims that we have to reverse my view on timing, arguing that top-down, strategic processes may be fast and bottom-up processes are slow, only playing a role later in processing. Even though intriguing, I think that there is little evidence for this radical view. We adhere to the position that bottom-up and history- based effects are fast, while top-down volitional control is typically (but not always) relatively slow.

*Its scope:* Some commentators downplay the role of selection history in attentional selection. For example, in his commentary, Wolfe indicates that selection history basically plays no role outside the lab, where search targets may be highly variable: “*an effective account of how we make our way in the world will require a greater appreciation of our relatively slow, volitional choices*” (p. 2). Similarly, Sisk et al. indicate that “*top-down control remains a cornerstone of visual selection*” (p. 1). Others stress the importance of selection history. Kryklywy and Todd underline its importance and argue that long-term life history (particularly outside the lab) dynamically tunes what they have called the “priority landscape”. Chelazzi and Santandrea point out that selection history is “*an instrumental tool for maximizing fitness to the environment which is implemented through plastic changes in critical neural substrates*” (p. 2). Concurring with these latter commentators, I think that the importance of selection history as a driving force is large, crucial and is omnipresent in our daily life. It is indeed a tool for maximizing fitness to the environment. It is an illusion to think that in everyday life we rely on top-down volitional choices. Instead, unconsciously the human brain continuously generates predictions about the environment based on our experiences and learned regularities. In turn, these regularities drive visual selection in an efficient, effortless way above and beyond any top-down and bottom-up processes (Failing & Theeuwes, 2017; Wang & Theeuwes, 2018a, b).

## Data Accessibility Statement

The author has no data accessibility statement to declare.
